# The relevance of female overweight in infertility treatment: a position statement of the Italian Society of Fertility and Sterility and Reproductive Medicine (SIFES-MR)

**DOI:** 10.1007/s10815-024-03379-0

**Published:** 2025-02-04

**Authors:** Andrea Roberto Carosso, Alessandro Conforti, Danilo Cimadomo, Valentina Spadoni, Carlotta Zacà, Claudia Massarotti, Alberto Vaiarelli, Roberta Venturella, Amerigo Vitagliano, Andrea Busnelli, Mauro Cozzolino, Andrea Borini, on behalf of the Italian Society of Fertility, Sterility, Reproductive Medicine (SIFES-MR)

**Affiliations:** 1 Obstetrics and Gynecology 1U, Physiopathology of Reproduction and IVF Unit, Department of Surgical Sciences, Sant’Anna Hospital Città della Salute e della Scienza di Torino, University of Torino, Via Ventimiglia 1, 10126 Turin, Italy; 2 Department of Neuroscience, Reproductive Science and Odontostomatology, University of Naples Federico II, Naples, Italy; 3 IVIRMA Global Reseach Alliance, Genera, Clinica Valle Giulia, Rome, Italy; 4 IVIRMA Global Research Alliance, 9.baby, Bologna, Italy; 5 IRCCS Ospedale Policlinico San Martino, Genova, Italy; 6 Department of Neurosciences, Rehabilitation, Ophthalmology, Genetics and Maternal and Child Health (DINOGMI Department), University of Genova, Genova, Italy; 7 Unit of Obstetrics and Gynecology, University of Catanzaro “Magna Grecia”, Catanzaro, Italy; 8 First Unit of Obstetrics and Gynecology, Department of Interdisciplinary Medicine (DIM), University of Bari, Bari, Italy; 9 Department of Obstetrics and Gynecology, IRCCS Humanitas Research Hospital, Milan, Italy; 10 Department of Biomedical Sciences, Humanitas University, Pieve Emanuele, Milan, Italy; 11 IVIRMA Global Research Alliance, IVI Roma, Rome, Italy; 12 IVIRMA Global Research Alliance, Fundación IVI-IIS la Fe, Valencia, Spain

**Keywords:** ART, BMI, IVF, Female obesity, Overweight, Age, Preconception counseling

## Abstract

**Purpose:**

Obesity is increasingly at the center of modern international healthcare systems. This is a position statement of the Italian Society of Fertility and Sterility and Reproductive Medicine (SIFES-MR) aimed at evaluating the impact of female overweight on infertility in order to improve fertility outcomes, including Assisted Reproductive technology (ART) treatments.

**Methods:**

The SIFES-MR writing group for this position statement was composed by Italian reproductive physicians, embryologists, and scientists with expertise in fertility evaluation, assisted reproduction technologies, and laboratory quality management. The positions stated are based on consensus by the authors, who met over a six-month period. The consensus emerged after thorough review of pertinent literature and standards concerning the impact of female overweight, complemented by extensive dialogue and discussion among the authors. Additionally, input from society members was considered, leading to revisions and eventual approval by the SIFES-MR governing council.

**Results:**

An increasing number of women affected by overweight and infertility accessing to ART treatments are expected in the future. A comprehensive counseling since the first access to infertility care is mandatory and should promote weight restoration, with the aim to improve the likelihood of spontaneous unassisted conception. Careful preconceptional evaluation of obese women is strongly encouraged for counseling purpose and comorbidities should be corrected by a multidisciplinary approach before spontaneous or medically assisted conception. Indeed, female obesity is responsible for high-risk pregnancies, with potential consequences in infants and during childhood. When in vitro fertilization is indicated, the risk of venous thromboembolism exacerbated by controlled ovarian stimulation should be assessed.

**Conclusions:**

Before IVF, different therapeutic approaches and expectant management to reduce overweight could be offered, and the age-related algorithm herein proposed by SIFES may represent an interesting tool for a better personalization of infertility care in these women. The treatment of infertility cannot ignore the correct management of female overweight, given the serious consequences that this condition can have on the outcomes of pregnancies and future generations. IVF specialists should tailor access and modalities of IVF treatment to this class of high-risk women.

## Introduction

In 2022, the World Health Association (WHO) report that approximately 390 million adults aged 18 years and older were underweight, while 2.5 billion were overweight, including 890 million who were living with obesity. In particular, obesity is an increasingly prominent focus of current healthcare costs, policies, and interventions [1]. The latest WHO update shows that, since 1975, global obesity rates have almost tripled. Data also show that over 340 million children and adolescents aged between 5 and 19 years were overweight or obese in 2016 [2]. Generally, women have higher rates of obesity than men, and in European countries overweight and obesity have reached epidemic proportions, affecting almost 60% of adults [3]. Recent evidence reports that obesity in women is associated with a wide range of gynecological disorders, including infertility. This condition can have early origins, as shown by the Childhood Determinants of Adult Health (CDAH) study, including more than 1500 schoolgirls followed for 25 years to evaluate whether childhood obesity is associated with infertility in women’s reproductive-aged life. The results demonstrated that childhood obesity before 12 years of age appears to increase the risk of female infertility in later life [4]. Considering the WHO report in 2016, 340 million children and adolescents may be at risk of attending fertility and pregnancy services in the foreseeable future. Despite current progresses in infertility care and in vitro fertilization (IVF) techniques, several adverse reproductive outcomes such as poor fertilization, abnormal embryo development, and poor offspring growth and vulnerability to disease have been associated to infertile obese women [1], with important psychosocial and economic implications. Indeed, the ability to have children is considered the social norm in many societies and couples affected by infertility may be subject to mental health issues including depression. Furthermore, obese women have a lower chance of live birth per cycle which may invariably affect cost and accessibility to treatments [2].

Although the impact of overweight and obesity on infertility care is a topic of great debate, guidelines on the treatment of this class of patients are weak.

The aim of this position statement is to evaluate the impact of female overweight on infertility, and to give practical indications for the management of this condition in order to improve fertility outcomes, including assisted reproductive technology (ART) treatments.

## Material and methods

This is a position statement presented on behalf of the Italian Society of Fertility, Sterility and Reproductive Medicine (SIFES-MR) by a group of its members. The writing group includes Italian reproductive physicians, embryologists and scientists with expertise in fertility evaluation, assisted reproduction technologies and laboratory quality management.

The positions stated are based on consensus by the authors, who met over a six-month period, as well as society member consultation with revisions and final approval from the SIFES-MR governing council. Consensus was achieved through review of relevant literature and standards related to overweight impact and management on infertility treatments with dialogue and discussion by the authors.

The main outcome of this position statement is to review the impact of weight disorders and provide an ideal framework before and during infertility therapies.

### International and national scenario

A comprehensive analysis of guidelines and recommendations from leading reproductive medicine societies reveals a nuanced approach to addressing obesity’s impact on fertility and reproductive treatments (Table 1).

**Table 1:** BMI and reproduction: existing guidelines and other scientific societies’ documents

Society	Guidance
American Society of Reproductive Medicine (ASRM) [5]	- Obesity/BMI should not be the sole criteria to deny reproductive treatments - The safety of oocytes retrieval in women with obesity should be evaluated multidisciplinary - In anovulatory women, weight loss increases the chances of spontaneous conception. However, in ovulatory women, it does not increase the success rates of ART - Prepregnancy counseling for couples with obesity should address the possible obstetrics risks associated with obesity - Additional research is needed to further the understanding of the effect of obesity on male reproductive function
European Society of Human Reproduction and Embryology (ESHRE) [6]	- No mention of BMI cut-offs or intervention to reduce BMI before ovarian stimulation -States that BMI does not predict ovarian response
Canadian Fertility & Andrology Society (CFAS) [7]	- Advices screening for comorbidities such as diabetes, hypertension and dyslipidemia before conception - Discusses evidence for the following complications linked to obesity: reduced spontaneous fertility, lower oocyte yield with ART, increased risk of pregnancy loss, lower live birth rates with egg donation, decreased safety during oocytes retrieval - Discusses evidence for the following Obstetrics complications linked to obesity: increased risk of gestational diabetes and macrosomia, preeclampsia, prolonged first stage of labor, instrumental deliveries, shoulder dystocia and cesarean section - All clinics with BMI cut-offs should also offer resources to obtain a healthy weight loss - Diet and exercise should be the first-line treatment. When weight loss surgery is needed, the woman must wait 1–2 years to conceive
Scottish Government [10]	BMI of female partner above 18.5 and below 30 before is an access criterion for ART
National Institute of Health and Care Excellence (NICE) [8]	- Women with a BMI of 30 or over should be informed that they are likely to take longer to conceive - States that female BMI should ideally be in the 19–30 range before ART, but does not advice for a restrictive access criterion - Men with a BMI of 30 or over should be informed that are likely to have reduced fertility
British Fertility Society [9]	- Women should aim for a normal BMI before starting any form of fertility treatment: ideally ART should be deferred until BMI is less than 30 (less than 35 in women with reduced ovarian reserve) - Patients should be provided with assistance to lose weight - At least patients should aim for a moderate weight loss of 5–10% of body weight, that could alone restore fertility - The couple must be informed of the increased risk of maternal, fetal and neonatal complications linked with obesity - BMI is am easy and reproducible measurement, but waist circumference and more detailed measures of metabolic risk are advisable in patients at high risk of insulin resistance

*BMI* body mass index, *ART* assisted reproduction technologies

The American Society of Reproductive Medicine (ASRM) advocates against sole reliance on Body Mass Index (BMI) in denying treatments, stresses multidisciplinary evaluation to ensure for safety in oocyte retrieval, and recommends a detailed preconception counseling regarding the higher obstetrics risks in obese pregnant women [5].

The European Society of Human Reproduction and Embryology (ESHRE) does not have a dedicated guideline regarding weight disorders and fertility treatments. The ESHRE clinical guideline: “Ovarian stimulation for IVF/ICSI” mentions BMI only to state that does not predict ovarian response [6]. Coherently, also the Canadian Fertility & Andrology Society (CFAS) does not emphasize BMI cut-offs for interventions before ovarian stimulation, but highlights obesity’s association with reduced fertility and increased obstetric complications, recommending early screening for comorbidities and emphasizing diet and exercise as primary interventions [7].

In contrast, both the National Institute of Health and Care Excellence (NICE) and the British Fertility Society (BFS) lean toward deferring assisted reproductive technology until achieving a BMI under 30 kg/m^2^, despite not mandating restrictive criteria. NICE introduces the notion that obesity alone might reduce fertility in both men and women [8]. These guidelines stress the risks of maternal and fetal complications, advocating for a 5–10% pretreatment weight loss [9]. Despite the absence of a strong recommendation for fixed restrictive criteria based only on BMI in most of available guidelines, in certain contexts, such as government-reimbursed cycles, national health care systems adopt BMI as a restrictive criterion, as seen in the Scottish government's document limiting in vitro fertilization (IVF) access to women with a BMI above 18.5 and below 30 [10]. To date, there is no national guideline that limits access to ART on the basis of BMI in Italy. The scenario is heterogeneous, and some regions adopt a BMI limit of 32 for access to IVF techniques, justifying this choice with a view to preventing serious obstetric complications in this class of patients. Surprisingly enough, much less attention is given to underweight and, to the best of our knowledge, no limitations are proposed to underweight women before IVF.

Despite divergent approaches, these viewpoints underscore the significance of addressing weight disorders and, in particular, obesity’s impact on fertility while highlighting the intricate balance between reproductive outcomes and weight management in clinical practice.

### Impact of female overweight on natural conception

Studies have shown that both lower and higher BMI levels can negatively affect spontaneous fertility. Obesity, in particular, has a profound impact on reproductive health as these women are at increased risk for ovulatory dysfunction, infertility, and pregnancy-related complications [11]. This is true also for metabolically healthy obesity, a subset of obesity with no metabolic abnormalities [12]. Obesity is associated with hormonal imbalances including insulin resistance, hyperinsulinemia, low sex hormone-binding globulin levels, elevated androgen levels, increased conversion of androgens to estrogens in peripheral tissues, higher free insulin-like growth factor 1 levels, and elevated leptin. The combined effect of these alterations may cause hypothalamic dysfunction, aberrant gonadotropin secretion, reduced folliculogenesis, and lower luteal progesterone levels [13, 14]. It has been observed that women with excess abdominal fat, indicated by a higher waist circumference, are more likely to experience anovulation compared to obese women with the same BMI but lower abdominal fat. This highlights the importance of body fat distribution in relation to anovulation [15]. Indeed, dysregulated adipocytes of upper body fat are resistant to the antilipolytic effects of insulin, resulting in the shunting of postprandial free fatty acids to the skeletal muscles and the liver. This fat storage worse insulin resistance [16] that with hyperandrogenemia are pivotal mechanisms through which obesity and fat distribution lead to anovulation. Furthermore, a number of molecules produced by fat cells have been involved in follicular function and ovarian steroidogenesis. In particular, abdominal obesity has been associated with reduced antioxidant activity within follicular fluid [17], potentially contributing to a deterioration of folliculogenesis and ovulation.

Obesity may also alter the endometrium and endometrial gene expression during the implantation window of natural cycles in obese women [18]. Some molecules involved in endometrial receptivity have been previously described altered in women with excess body weight; in particular, a negative correlation between endometrial glandular leukemia inhibitory factor (LIF) concentration and BMI and a different endometrial protein profile due to an increased expression of haptoglobin have been reported [19, 20]. This last mechanism could explain why miscarriage risks are also increased in women with obesity, who conceive spontaneously. A Danish cohort study involving over 5000 women reported a hazard ratio for miscarriage of 1.23 in obese women compared to non-obese controls [21]. Similar results were reported in other cohorts, reporting higher risk of early and recurrent early miscarriage in obese women [22], as confirmed by a quite recent systematic review and meta-analysis [23]. Interestingly enough, an increased in miscarriage rate is observed not only in obese women but also in every BMI outside of the normal category [24]. Several mechanisms behind the link between overweight and recurrent miscarriage have been proposed: an increased endometrial expression of haptoglobin, transthyretin, and beta-globulin, which are inflammatory factors [20]; higher serum leptin levels, which may alter endometrial epithelium receptivity [25]; the state of relative hyper-estrogenemia (due to aromatization of androgens to estrogen in adipose tissue) and its detrimental effect on endometrial receptivity [26, 27].

These issues become even more pronounced with advancing age, particularly in women at the age of 35 years or older (advanced maternal age (AMA)) [28]. Indeed, several studies suggest that advancing age at the time of pregnancy is associated with greater disparities in severe maternal morbidity and mortality, and the combined effect of AMA and either overweight or obesity increase reproductive risks [29].

### Impact of female overweight on assisted reproduction

Within the context of ART, the maintenance of women’s BMI within a normal range represents a crucial element in optimizing IVF and obstetrics outcomes. The association between body weight and cumulative live birth rates after IVF outcome follows an “inverted U shape” [30] implying that the manifestation of both underweight and overweight/obesity can have deleterious effects on IVF outcomes [31–34].

The ESHRE guideline on ovarian stimulation notes that BMI alone is not a reliable predictor of ovarian response. Despite this, only a limited number of studies have evaluated the accuracy of BMI in this context [6]. One important area of investigation is the impact of BMI on gonadotropin resistance, which refers to the suboptimal response to gonadotropins during ovarian stimulation. Interestingly, recent research has shown a significant correlation between BMI and the threshold for exogenous follicle-stimulating hormone (FSH) administration; specifically, higher BMI levels are associated with an increased dosage for FSH. The clinical evidence of the aforementioned relation between BMI and gonadotropin resistance is provided by some studies demonstrating that the dosage of gonadotropins tends to be higher in women with a BMI over 30 kg/m^2^ compared to those with lower BMI [35]. Notably, the dose of gonadotropins appears to be a crucial factor, as it correlates with the ovarian response, influencing the success of ovarian stimulation procedures and of the whole IVF treatment. Several factors may contribute to this gonadotropin resistance in women with elevated BMI. First, individuals with a higher BMI typically have a greater body surface area, which requires a larger amount of exogenous FSH. Additionally, changes in absorption and metabolic clearance, along with lower levels of sex hormone-binding globulin (SHBG), can alter peripheral steroid metabolism, further contributing to gonadotropin resistance [36].

Furthermore, the Ovarian Sensitivity Index (OSI), which represent the ratio between the number of retrieved oocytes/total gonadotropin dose [37], is significantly associated with age and BMI, displaying an inverse relationship. Lastly, the Anti-Müllerian Hormone (AMH) and Antral Follicle Count (AFC) which are commonly used to predict gonadotropin starting dose of COS exhibit a direct correlation with BMI [38, 39].

This intricate interplay between BMI and ovarian stimulation may suggest the importance of tailoring stimulation protocols based on the patient’s BMI to enhance treatment efficacy [40] and minimize potential complications during ovarian stimulation procedures [41].

In the setting of ovarian stimulation, the antagonist protocol seems more effective for women with normal or elevated BMI than GnRH agonists. The principal advantages include a lower requirement for recombinant FSH, a reduced probability of Ovarian Hyperstimulation Syndrome (OHSS), a shorter duration of ovarian stimulation, and a decrease in patient discomfort.

Recognizing BMI’s impact beyond ovarian stimulation is crucial because scientific evidence consistently highlights BMI’s notable effects on several aspects, including oocyte quality, embryo development, and blastulation rate. This extends beyond mere quantity, as maternal age and BMI jointly exert significant adverse causal effects on the rate of metaphase II (MII) oocytes in patients undergoing ART, adjusting for confounders. Regardless of the protocol used, BMI influences the number of MII oocytes retrieved, with obesity significantly increasing the likelihood of cycle cancelation [42, 43].

Furthermore, abnormalities in oocyte morphology are observed in women with a high BMI, where oocytes from overweight/obese women are smaller than those from women within a healthy BMI [44, 45]. Maternal obesity introduces alterations to the ovarian follicular environment, impacting oocyte developmental competence [46]. This includes changes in adipokine signaling, lipid content, mitochondrial dysfunction leading to oxidative stress, and alterations in the epigenetic signature. These factors collectively impair oocyte quality and subsequent embryonic and fetal development. As a result, BMI’s impact extends to embryo competence [47].

Indeed, BMI might not impact the euploid blastulation rate per MII [48], but it influences reproductive outcomes, because women with higher BMI showed higher miscarriage after euploid embryo transfer than women with normal BMI [32, 49–51] . However, clinical miscarriages seem to increase in women with obesity without alteration in biochemical miscarriages than in women with normal weight [44]. These findings suggest that higher miscarriages in women with obesity are not related to aneuploidies, opening up to interesting alternative scenarios involved in oocyte and embryo competence, such as mitochondria, which have been shown to be deeply affected by unbalanced dietary pattern leading to overweight phenotypes [52] or to altered ovarian leptin signaling [53].

Anyway, a higher clinical miscarriage rate constitutes the main determinant of the significant reduction in live birth rate observed in women with obesity with chromosomally normal fetuses [51]. Among the possible explanations in addition to speculation on oocyte competence, a pivotal role is attributed to altered endometrial function in overweight women [54]. In the context of fresh embryo transfer, elevated estradiol levels observed in women with high BMI may contribute to endometrial alterations, because of a more robust and prolonged stimulation than women with a regular BMI. These supraphysiological hormonal levels could potentially modify endometrial ultrastructure and consequently unpair the embryo-endometrium synchrony [55, 56]. Other studies have pointed to underlying molecular mechanisms related to the abnormal expression of endometrial genes that regulate the process of embryonic implantation and to metabolic disturbances associated [18, 56]. Recently, an analysis of more than 56,000 euploid blastocysts showed that increasing BMI was associated with decreased live birth rate and increased pregnancy loss. However, these results were attenuated among patients with a sole diagnosis of male factor infertility, suggesting that associated metabolic disturbances, as for PCOS women, and not BMI alone may underlie this trend [57].

Finally, weight disorders may have an impact on complications of transvaginal ultrasound-guided oocyte pickup (OPU), particularly in severe obese women. To date, no increased risk of complications for women with obesity undergoing OPU with conscious sedation have been reported. However, difficult access and incomplete aspiration of follicles through a transvaginal approach were more likely to be encountered in patients with elevated BMI. Indeed, compared with patients with BMI < 25 kg/m^2^, obese women were more likely to require additional sedation during the procedure; the rate of difficult access was 28.9% for procedures in obese women compared with 5.2% with BMI < 25 kg/m^2^, and the OPU was incomplete due to inaccessible follicles through a transvaginal approach in 18.2% of procedures of obese women compared with 1.3% with BMI < 25 kg/m^2^ [58].

Overall, from a clinical perspective, a woman’s BMI should not be viewed merely as a factor influencing ovarian stimulation; rather, it should be regarded as a comprehensive determinant that shapes critical aspects of the entire assisted reproduction process.

### Impact of female overweight on egg donation treatment

Several authors speculated that recipient’s obesity could impair the reproductive outcomes of IVF with donor oocytes by altering the endometrial gene expression in the peri-implantation period. Several studies investigated this issue and obtained conflicting results [59].

Jungheim et al. conducted a systematic review and meta-analysis including 8 and 5 studies in the qualitative and quantitative analysis, respectively. No associations between obesity and chance of pregnancy after IVF were observed in women using donor oocytes. Additional analyses assessing associations between recipient obesity and embryo implantation, miscarriage and live birth also failed to show a negative effect [60]. More recently, Setton et al. analyzed the relationship between BMI and endometrial receptivity by investigating the outcomes from sibling-oocyte recipients from the same donor egg cycle. Through this idealized model, oocyte quality was controlled to a greater degree than in any prior study in order to explore the impact of BMI on endometrial receptivity. Reproductive outcomes (i.e., implantation rate, pregnancy rate, miscarriage rate, and live birth rate) between normal weight and overweight/obese women did not differ [61]. Similar results have been reported elsewhere [62].

However, the results of two more recent studies are in contrast with previous findings. Specifically, a raised recipient’s BMI was associated with a reduction in cumulative live birth rate [63] and higher miscarriage rate per clinical pregnancy. In contrast, underweight does not appear to have similar effects [64].

Regardless of the impact on success rates, ideally, the recipient’s BMI values should be in the normal range before undergoing an IVF cycle with donated eggs. In fact, a normal BMI has undisputed benefits both on the general health of the recipient and on that of the pregnancy. In this specific context, however, the time needed to achieve a reduction in BMI is a pivotal issue. In fact, there is also no doubt that advancing maternal age, is associated with an increase in the rates of severe obstetric and neonatal complications. This increase becomes extremely considerable from the age of 45 onwards [65]. Clinicians should consider both aspects in order to achieve the optimal balance for each patient, taking in account that there is a strong association between extremity maternal body mass index and an increased risk of metabolic (obesity, type 2 diabetes, cardiovascular disease, and metabolic syndrome) and nonmetabolic (cancer, osteoporosis, asthma, and neurologic alterations) diseases in the offspring, probably mediated by epigenetic mechanisms of fetal programming [66, 67].

## Discussion

Considering that more and more couples who refer to IVF center are affected by weights disorders, and overweight in particular [5], an appropriate multidisciplinary counseling is required since the first approach. The first aim is to identify and treat comorbidities related to overweight before undergoing medically assisted treatment. Indeed, obese women are at risk of develop hypertension, metabolic syndrome, diabetes, insulin resistance, and venous thromboembolism (VTE). VTE is considered a severe complication of IVF procedure [68], and such risk could be potentially exacerbated in obese women. Thus, we recommend a proper evaluation of VTE risk should be carried out in obese women before IVF procedure. According to RCOG guideline, obesity (BMI ≥ 30 kg/m^2^) together with medically assisted reproduction treatments and OHSS represent a crucial risk factor that need to be considered by clinician to tailor the prescription of low-molecular-weight heparin (LMWH) in order to prevent severe thromboembolic event during pregnancy. In details, the presence of these three risk factors and any other proposed additional factor (as age > 35 years, see Table 1 of RCOG guideline) makes the woman candidate for the use of (LMWH) antenatally [69].

Apart from IVF treatment, restoration of a healthy weight should be pursued even before spontaneous attempts. Indeed, women with BMI above 30 kg/m^2^ should be informed about the potential risk during pregnancy such as miscarriage, gestational diabetes, hypertensive disorders, venous thromboembolism, post-partum hemorrhage, and mortality [70]. Even infants of obese mother show an increased risk to develop congenital anomalies, stillbirth, prematurity, and macrosomia. Several prospective studies have also demonstrated long-term adverse metabolic consequences in childhood and adulthood [70].

An appropriate counseling should be supported by the collaboration of specialized professional figure. For instance, the presence of nutritionists in IVF center could sustain the care of obese patients before IVF treatment and during the eventual pregnancy not simply aimed at losing weight but especially in improving metabolic health [71]. It should be also stressed that obese women need specific nutritional support during pregnancy. For instance, women with BMI above 30 kg/m^2^ need higher amount of folic acid supplementation comparing with women with normal weight (5 mg folic acid at least 1 months before conception until the first trimester of pregnancy) [72]. Furthermore, obese women and their neonates are at increased risk of Vitamin D deficiency [73].

The support of endocrinologists specialized in the management of pharmacological treatment of obesity should be requested, as emerging evidence shows the benefits of glucagon-like peptide-1 receptor agonist for managing insulin resistance associated with polycystic ovary syndrome. Although to date there is no data on the benefit of such treatments before ART, a combined treatment with glucagon-like peptide-1 receptor agonist and metformin has been shown to have significant effects on weight loss and favorable results on endocrine and metabolic parameters, in women diagnosed with polycystic ovary syndrome and obesity or overweight [74].

Lastly, bariatric surgery should be considered in patients who have not benefited from the aforementioned interventions. Even if there is a paucity of literature on the impact of bariatric surgery on ART outcomes, a systematic review and metanalysis show that bariatric surgery prior to ART may have an impact on cumulative live birth rates with fewer pregnancy-related complications [75].

Further studies on the nutritional, pharmacological, and surgical intervention of infertile women are mandatory to define decision-making algorithms that can improve the reproductive outcomes of these patients.

Another issue in the evaluation of overweight women is fat distribution. Although BMI is commonly used also in research to define and classify obesity, it has a high specificity but a low sensitivity to detect adiposity, and it fails to identify nearly half of women with excess fat mass [76]. It has been estimated that normal weight individuals could have abnormal metabolic profiles and be at increased risk of developing obesity-associated diseases. These individuals are the so called normal weight-obese women showing a BMI in the range of normal weight but a fat percentage > 30% [77, 78].

BMI cannot differentiate lean from fat mass neither body fat distribution. Central body obesity is associated with a major increase in metabolic and cardiovascular risk, hyperinsulinemia, and insulin resistance [79, 80], so particular attention should be directed to these patients. For instance, waist circumference was inversely related to the probability of live birth among women undergoing assisted reproductive technology independently of body mass index [81]. In the future, anthropometric measures other than BMI related to IVF and obstetrics outcomes should be evaluated, as individuals with the same BMI may have different adipose tissue distribution, body composition and consequently different risk profile. Waist circumference (WC), hip circumference (HC), and waist-to-hip ratio (WHR) seem to be the most promising.

### Management of female overweight: the SIFES algorithm

In Italy, there is no BMI threshold for the access to fertility treatment. In other countries, several BMI thresholds have been proposed by International Fertility Society, as discussed above.

Negate IVF procedures solely on the basis of women BMI without considering the age-related fertility decline could be inappropriate. Indeed, while in young patients, the IVF procedure could be postponed without substantial consequences; in advanced years old women excessive delay could significantly impair the chance of success. Indeed, it was widely demonstrated than fertility rate above 35 years old declines dramatically years by years [82–84]. Furthermore, age has a greater impact than BMI on IVF success, particularly in women of advanced age [85]. On the other hand, IVF procedure in women with BMI over 35 kg/m^2^ could increase the risk of thrombotic event during ovarian stimulation and could have important consequences during pregnancy. In this sense, irrespective of women age, a comprehensive counseling since the first access to IVF center should be pursed with the aim of making women aware of the risks of achieving pregnancy with elevated BMI and the possible benefits of a dietary approach before conception.

We recognize that basing infertility treatment decisions on weight raises significant bioethical discussions of considerable interest. While it is true that not all overweight women are infertile, and therefore denying access to conception may seem ethically unacceptable, it is clearly demonstrated that this condition alone can be responsible for infertility. As reproductive medicine specialists, it is therefore legitimate to ask ourselves whether our first objective should be the treatment of the causes of infertility and the restoration of natural fertility, or the use of IVF, potentially able of directly delivering to the couple “a child in arm.” From this perspective, we believe that a balance between these two policies should be promoted, and the most appropriate tool to guide this choice is female age, because this represents the best predictive factor of women's future reproductive potential [86].

**Fig. 1:**
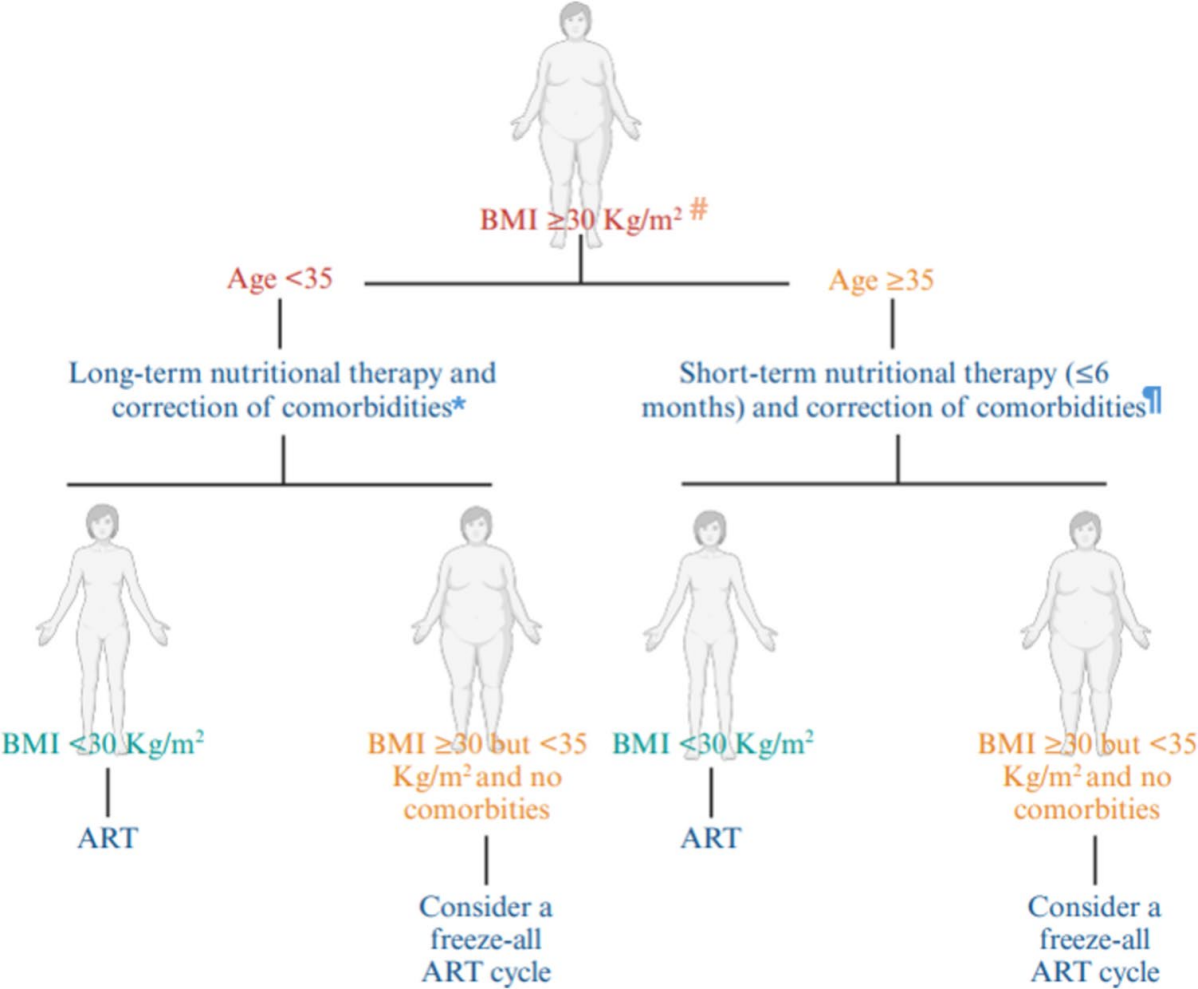
Age-related algorithm proposed by Conforti et al. on behalf of SIFES. Legend: # apart from BMI, other measures such as waist circumference or waist-to hip ratio need to be evaluated especially in sporty or skinny fat women; *consider bariatric surgery as second-line approach; ^¶^consider bariatric surgery as first-line approach in case of severe obesity

In this position statement, we proposed a different approach for obese women based on the age which still represent the most relevant parameter related with IVF success. The age-related algorithm proposed by Conforti et al. on behalf of SIFES (Fig. 1) could represent an interesting tool for clinician to manage women with obesity characterized by normal ovarian reserve. Indeed, women with diminished ovarian reserve present reduced prognosis to IVF procedures irrespective of age [87, 88], and their management is outside the scope of this paper.

In young women (age below 35 years), diet and nutritional/pharmacological/surgery approaches are strongly recommended because they could improve the chance of spontaneous conception especially in couple with no other main causes of infertility [89]. Furthermore, weight loss could significantly improve obstetric outcome of IVF pregnancies. On the other hand, when the couples need medically assisted treatment, ovarian stimulation should not be procrastinated above 35–37 years old. For instance, in absence of comorbidities and anesthetic concern, the oocyte retrieval could be safely carried out even in women with class I obesity or more [5]. Nonetheless, oocyte retrieval in obese women should be avoided in outpatient setting without appropriate anesthetic facilities. IVF procedure in advanced reproductive age women with BMI between 30 and 35 kg/m^2^ could be proposed in the absence of comorbidities or anesthetic concerns. Above 35 kg/m^2^, despite some encouraging data [90], oocyte retrieval should be avoided until the achievement of weight loss (BMI ≤ 35). In addition, in severe obese conditions, the anesthetic risk and obstetric complication become probably too relevant.

Despite the fact that we recognized that WC and WHR could reflect better abdominal fat distribution, very few studies analyzed IVF outcomes considering these parameters. On the other hand, BMI was widely investigated in ART setting and so it was considered the main parameter on which to build the present algorithm. However, WC below 88 cm (35 inches) and WHR below 0.85 are desirable goals in infertile women to reduce the risk of metabolic disorders, in line with WHO recommendation [91, 92]. Furthermore, WC and WHR become more relevant in particular subgroup of patients, such as sporty women (e.g., body builders) or skinny fat women in whom BMI could be deceptive [93]. In particular, sporty women could have a misleading elevated BMI due to their high muscle mass. These women should not be subjected to interventions aimed at reducing BMI as they are not at metabolic risk, and therefore WC and WHR might be considered as “pilot” anthropometric parameters for every clinical decision. In contrast, skinny fat women may benefit from nutritional and lifestyle interventions due to their increased abdominal adiposity, despite a normal BMI range.

In addition to the nutritional and lifestyle interventions provided by our algorithm, bariatric surgery needs to be considered. Given that the delay of pregnancy is recommended to prevent fetal exposure to nutritional deficiency [94], even bariatric surgery needs to be tailored according to patients age. Indeed, in more time-sensitive cases, such as women in advanced reproductive age, bariatric surgery could be considered the first-line approach in severe obesity when the diet is unlikely to yield short-term results. On the other hand, in young women (below 35 years old), it could be considered mainly as a second-line approach only when long-term nutritional approach failed.

The present algorithm could not be fully applied in women with obesity affected by specific fertility-related disorder such as PCOS or endometriosis, the management of which has already been addressed in the dedicated international guidelines [95, 96].

## Conclusions

It is foreseeable that specialists in reproductive medicine will face an increasing number of women affected by overweight in the future.

A comprehensive and multidisciplinary counseling since the first access to IVF center is mandatory and should have the following goals:

- Weight restoration could improve the likelihood of spontaneous unassisted conception.
- Obesity presenting with comorbidities should be corrected by a multidisciplinary approach before spontaneous or medically assisted conception.
- IVF procedures including controlled ovarian stimulation are at increased risk of VTE that could be potentially exacerbated by the presence of obesity.
- Obesity is a crucial risk factor for important disorders during pregnancy and could have important consequences in infants and during childhood.
- Careful preconceptional evaluation of obese women is strongly encourage for counseling purpose and long-term management.
- The age-related algorithm proposed by SIFES might represent an interesting tool for a better personalization of IVF care in obese women.
- Apart from BMI, the measure of WC, HC, and WHR is strongly encouraged during the assessment of overweight and obese women for both clinical and research purposes.
